# Construction of tissue microarrays from prostate needle biopsy specimens

**DOI:** 10.1038/sj.bjc.6602726

**Published:** 2005-08-09

**Authors:** S Jhavar, C M Corbishley, D Dearnaley, C Fisher, A Falconer, C Parker, R Eeles, C S Cooper

**Affiliations:** 1 Sections of Molecular Carcinogenesis, Institute of Cancer Research, Sutton, Surrey SM2 5NG, UK; 2 Department of Cellular Pathology, St George's Healthcare Trust, Blackshaw Road, Tooting, London SW17 0QT, UK; 3 Department of Academic Urology, Royal Marsden NHS Foundation Trust and Institute of Cancer Research, Sutton, Surrey SM2 5PT, UK; 4 Department of Histopathology, Royal Marsden NHS Foundation Trust, Fulham Road, London, SW3 6JJ, UK; 5 Section of Cancer Genetics, Institute of Cancer Research, Sutton, Surrey SM2 5NG, UK

**Keywords:** prostate, cancer, needle biopsy, tissue microarray, immunohistochemistry

## Abstract

Needle biopsies are taken as standard diagnostic specimens for many cancers, but no technique exists for the high-throughput analysis of multiple individual immunohistochemical (IHC) markers using these samples. Here we present a simple and highly reliable technique for constructing tissue microarrays (TMAs) from prostatic needle biopsies. Serial sectioning of the TMAs, called ‘Checkerboard TMAs’, facilitated expression analysis of multiple proteins using IHC markers. In total, 100% of the analysed biopsies within the TMA both preserved their antigenicity and maintained their morphology. Checkerboard TMAs will allow the use of needle biopsies (i) alongside other tissue specimens (trans-urethral resection of prostates and prostatectomies in the case of prostate cancer) in clinical correlation studies when searching for new prognostic markers, and (ii) in a diagnostic context for assessing expression of multiple proteins in cancers from patients prior to treatment.

Needle biopsy specimens are taken at the time of diagnosis from many human malignancies, including oral cancer, breast cancer, sarcomas, prostate cancer and lymph node masses (Bostwick, 1997; Morrow *et al*, 2002; Neville and Day, 2002; Cormier and Pollock, 2004; Jereczek-Fossa *et al*, 2004). In prostate cancer, for example, examination of needle biopsy specimens yields valuable information, including Gleason grade and the tumour extent, that facilitates decision-making on the appropriate treatment. A major current problem in prostate cancer is to predict the behaviour of early and potentially localised disease (Yao and Lu-Yao, 2002). Some cases may remain dormant for many years without progressing, while others will progress rapidly to metastases. It is therefore important to identify the patients who need to be treated and separate them from those who can be managed by active surveillance, thus sparing the latter of the adverse consequences of unnecessary treatment (Dearnaley and Melia, 1997; Parker, 2004). If tests are to be developed that will allow the prediction of clinical behaviour of patients diagnosed with prostate cancer, for example, following prostate-specific antigen (PSA) screening, it must be possible to perform the test on biological specimens obtained from the patients at the time of diagnosis, which would usually only include trans-rectal ultrasound (TRUS)-guided needle biopsy specimens, blood or urine.

Tissue microarrays (TMAs) that allow many hundreds of tumours to be analysed simply in a single experiment have proven to be very useful in high-throughput evaluation of prognostic markers for predicting the clinical behaviour of patients with prostate cancer (Dhanasekaran *et al*, 2001; Rhodes *et al*, 2003; Foster *et al*, 2004). At the moment, construction of TMAs in prostate cancer studies is however limited to tissue samples obtained from punched-out cores from paraffin blocks from trans-urethral resection of prostate (TURP) specimens and from radical prostatectomies. Battifora and Mehta (1990) described a Checkerboard method for producing tissue arrays as a forerunner to the core-based TMA method described by Kononen *et al* (1998). In this Checkerboard method, multiple (up to 100) chunks of formalin-fixed de-paraffinised or fresh normal or tumour tissue were reset in agar and then in paraffin wax in a Checkerboard pattern. This procedure is unsuitable for formalin-fixed needle biopsy specimens because, following dewaxing, the tiny specimens are easily lost and very difficult to orientate and re-embed vertically. The TMA procedure described by Kononen *et al* (1998) is also unsuitable for use with prostate needle biopsy specimens. The diameter of these needle biopsies, which are conventionally embedded horizontally in paraffin blocks, is less than 1.26 mm, the outer diameter of an 18-G needle, and their depth is further eroded after the block has been sectioned for diagnosis. Hence, cores obtained by the Kononen *et al* (1998) method would be extremely difficult to align in the recipient block, and at best would provide only a few slices. We present an approach for constructing TMAs from needle biopsy specimens that overcomes these difficulties.

## MATERIALS AND METHODS

### Selection of paraffin blocks containing prostate needle biopsies

**Figure 1:**
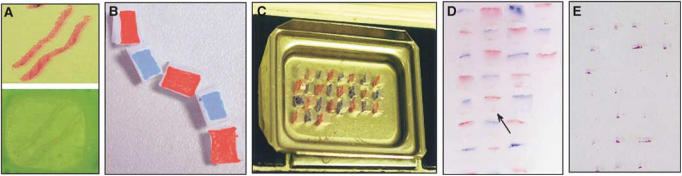
Construction of Checkerboard TMA from prostatic needle biopsies. (**A**) Prostatic needle biopsies embedded horizontally within a paraffin block (bottom) and H&E sections from this block (top). (**B**) Checkers (4 × 2 × 2 mm^3^) cut from the original paraffin block containing the prostatic needle biopsy specimen was painted on the surface opposite that containing the biopsy, alternately with blue and red dyes. (**C**) Checkers positioned in a 36 × 24 × 5 mm^3^ stainless steel mould (Raymond A Lamb Ltd, UK) so that each biopsy specimen now had a vertical orientation. (**D**) Finished Checkerboard TMA block in which the cross-sections of the vertically positioned biopsies are seen (arrow). (**E**) Haematoxylin and eosin-stained section (4 *μ*m) obtained from the Checkerboard TMA block.

Paraffin-embedded formalin-fixed prostate needle biopsy specimens (Figure 1A) were obtained from prostate cancer patients (i) who took part in the RT01 trial at The Royal Marsden Hospital NHS Trust and had given written consent for research to be undertaken on their biopsies (including their 2-year post radiotherapy biopsies) (*n*=2); (ii) who had diagnostic TRUS-guided prostate biopsies at the St Georges Hospital NHS Trust (*n*=23). The biopsies from the former group of patients were only used in the pilot experiments (data not shown). Biopsies from the latter group of patients were used in the validation set of TMAs and all patients in this group had died at the time of the study.

### Antibodies

The following antibodies were chosen: (i) low-molecular-weight keratin, CAM 5.2 (cat# 345779, supplied by Becton Dickinson, dilution 1 : 5), chosen to show all epithelial cells; (ii) high-molecular-weight keratin, LP34 (cat# M0717, supplied by DakoCytomation, dilution 1 : 50) that stains only basal cells and (iii) PSA (rabbit polyclonal, cat# A0562, supplied by DakoCytomation, dilution 1 : 20 000) that stains prostatic epithelial cells of acini and peripheral ducts.

### Immunohistochemistry (IHC)

Sections of the Checkerboard TMA were cut at 3 *μ*m onto Superfrost Plus™ positively charged glass slides (Menzel-Glaser, Germany), dewaxed with xylene and rehydrated to water through graded ethanol rinses. Endogenous peroxidase activity was blocked by immersion of tissue sections in a 10% (v v^−1^) solution of H_2_O_2_ in deionised water at room temperature for 8 min. In all, 1 l of diluted (1 : 10 in deionised water) Tris-EDTA-citrate (pH 7.8) buffer was preheated in a pressure cooker (Nordicware microwave tender cooker, Bio Genex) for 16 min without the slides. High-temperature antigen retrieval was then performed by placing the peroxidase-blocked slides in the hot buffer and sealing the pressure cooker, and comprised microwave (Panasonic 900 W) heating at high temperature for 6 min. After this, the pressure was released and cold water was allowed to run in the pressure cooker to cool the slides, which were now ready for staining. Chymotrypsin pretreatment was performed by placing the peroxidase-blocked slides in a chymotrypsin (Alpha Chymotrypsin Type II bovine pancreas, Sigma Cat# C-4129) solution in Tris buffer (pH 7.6) for 30 min. The slides were then washed by running tap water on them for 5 min. Staining after the primary antibody incubation involved the use of ChemMate Envision Kit K5007 from Dakocytomation, and the Optimax Plus Staining Machine (BioGenex, supplied by A Menarini) and manufacturer's instructions were followed. Sections were incubated with the primary antibody for 30 min at room temperature and washed with buffer (Optimax Wash buffer – BioGenex supplied by A Menarini Cat. No. HK583-5K).

## RESULTS

### Preparation of individual blocks or ‘checkers’

The starting point for construction of Checkerboard TMAs was paraffin donor blocks containing formalin-fixed prostate needle biopsy specimens embedded at their surface (Figure 1A). A scalpel blade was used to cut 4 mm lengths of biopsy specimens corresponding to areas of tumour or normal tissue identified from the original haematoxylin and eosin (H&E)-stained longitudinal section by the study histopathologist (CMC). Additional cuts were made in the wax to obtain a 4 × 2 × 2 mm^3^ cubical shaped block, which we called a ‘biopsy checker’, containing the 4 mm length of biopsy specimen attached on one of its 4 × 2 mm^2^ faces. To help orientate the block in later stages of the procedure, the 4 × 2 mm^2^ face opposite that containing the biopsy specimen was immediately painted with dyes – blue and red dyes were used to paint this face on alternate checkers (Figure 1B).

### Construction of the Checkerboard TMA

The checkers were then arranged in a square grid pattern in a 36 × 24 × 5 mm^3^ stainless steel mould (Raymond A Lamb Ltd, UK) (Figure 1C). The checkers were positioned in the mould so that each biopsy specimen had a vertical orientation allowing analysis of the cross-section of each biopsy. The mould was placed on the semiheated platform of the embedding apparatus, while the checkers were arranged. A predetermined template was used as a guide to position and record the identity of each checker within the array. A plastic cassette was then placed over the mould. The checkers were then re-embedded by pouring hot paraffin wax (60°C) into the mould through the cassette and the mould was then cooled by placing it on a cold platform (−5°C) for 1 h. Gentle rocking of the mould in a circular fashion was carried out while the hot paraffin wax was being poured to ensure that each checker was completely surrounded by wax. Sufficient care was taken to avoid formation of air bubbles in the wax between the checkers. The dyed surfaces of the checkers are monitored: (i) to mark the position of the checkers within the finished paraffin block attached to the plastic cassette and (ii) to ensure that the checkers remain in their correct orientation during the setting process. The alternate marking of individual checkers with blue and red dyes gave rise to a Checkerboard appearance (Figure 1D). Initial sections (4 *μ*m) cut onto Superfrost™ glass slides (Ultima) were stained by conventional H&E stains to identify the location and morphology of representative biopsy specimens within the microarray block (Figure 1E).

### Analysis of IHC markers using Checkerboard TMAs

Tissue microarrays were constructed from biopsies from deceased prostate cancer patients (*n*=23). In all, 45 checkers were prepared from needle biopsies from these 23 patients. Out of the 45 checkers, 41 retained their biopsy specimens in the Checkerboard TMA, equivalent to a tissue loss of 9%. The remaining checkers included 22 biopsy specimens that had regions considered to contain cancer based on the original H&E section of the horizontally embedded biopsy, and 19 that had been judged as normal.

**Figure 2:**
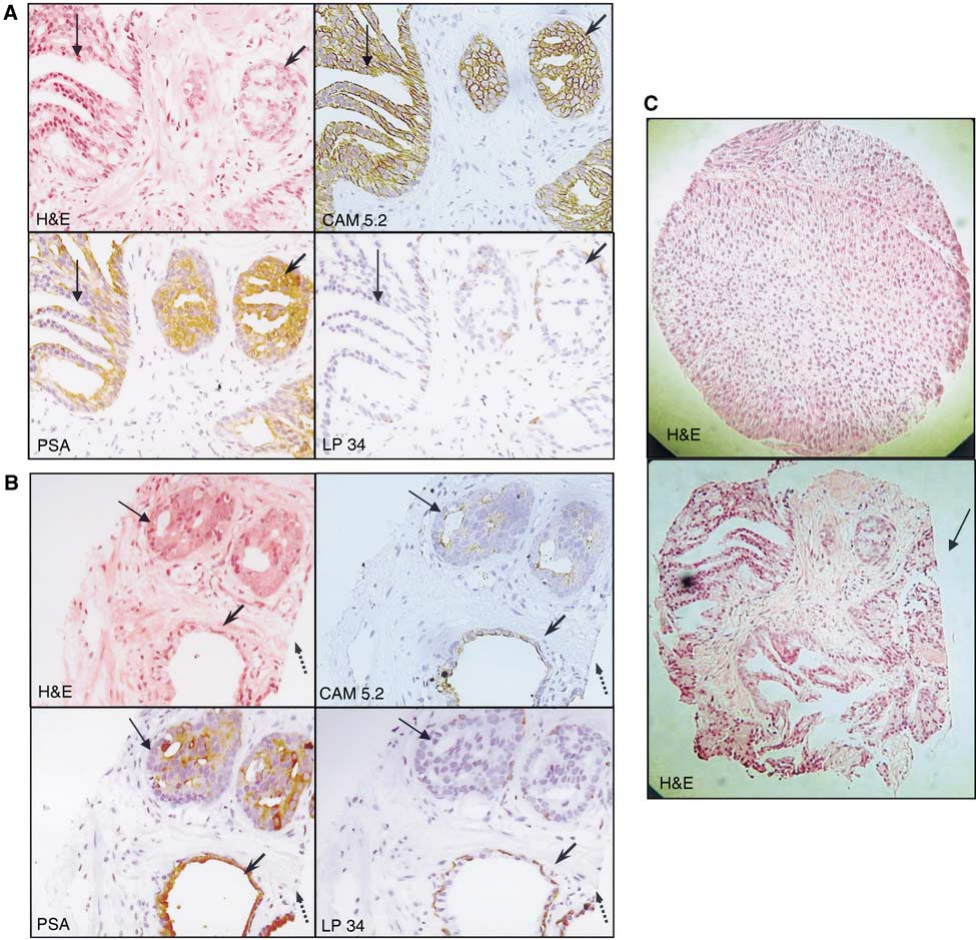
Analysis of sections obtained from a Checkerboard TMA. (**A**) Serial sections (× 20) from a single biopsy specimen that contained both cancer (thin arrow) and prostatic intra-epithelial neoplasia (PIN) (arrowhead) stained by H&E, PSA, CAM 5.2 and LP34. (**B**) Serial sections (× 20) of second biopsy specimen containing both cancer (thin arrow) in the deeper part of the biopsy and benign glands on the surface (arrowhead) stained by H&E, PSA, CAM 5.2 and LP3. The surface of the biopsy that was originally cut for diagnosis of cancer is seen (dotted arrow). (**C**) Comparison of a tissue core (× 20) from conventional TMA (top panel) with a biopsy core (× 20) from the Checkerboard TMA (lower panel). The side of the needle biopsy originally cut for diagnostic purposes can be seen (arrow).

Results of IHC studies on 123 individual tissue slices from the Checkerboard TMA demonstrated that antigenicity was preserved in 100% of the retained biopsies. Results from serial sections (3–4 *μ*m) of two representative biopsy specimens within the Checkerboard TMA containing cancer stained by H&E, PSA, CAM 5.2 (low-molecular-weight keratin) and LP34 (high-molecular-weight keratin) are shown (Figures 2A and B). The PSA, CAM 5.2 and LP34 antibodies are used routinely in the diagnosis of prostate cancer (Moll *et al*, 1982; Makin *et al*, 1984; Gillespie *et al*, 2002; Evans, 2003). An interesting feature of the Checkerboard TMA analyses was that cancer was identified in nine out of 19 (five out of 19 based on H&E staining alone) checkers containing biopsy specimens originally judged as normal.

A comparison of a section of core obtained from conventional TMA with that obtained from Checkerboard TMA method described in this report is shown in Figure 2C. The cross-sections are very similar in size, but the tissue specimen from the Checkerboard TMAs generally has a more irregular shape and the side of the needle biopsy originally cut for diagnostic purposes can usually be seen (Figure 2C, arrow).

## DISCUSSION

We have demonstrated that it is possible to construct TMA from diagnostic prostate needle biopsies. Prostate needle biopsies are formalin fixed and set horizontally on the surface of a paraffin wax block so that a lengthwise slice of the entire biopsy specimen can be taken for diagnostic examination. Our method for constructing TMAs from these specimens involves cutting out small 4 mm lengths of the biopsy specimen and repositioning them in a vertical orientation so that approximately 90–100 4-*μ*m-thick sections from each biopsy can be made available for IHC staining, in comparison to only a few from the original horizontally embedded specimens. Such formalin-fixed material could also, in principle, be used for other molecular procedures such as fluorescence *in situ* hybridisation (FISH) (Takahashi *et al*, 1994; Bastacky *et al*, 2004).

The IHC staining results from our study show that the biopsy tissue maintains its antigenicity even after embedding at the surface of the paraffin block and re-embedding the biopsy checkers in the Checkerboard TMAs. Using conventional TMA technology, Rubin *et al* (2002) concluded that less than three cores may not accurately represent protein expression, but that more than four cores were unnecessary and did not add to the value of arrays used to predict prostate cancer outcome. In the method described here, multiple assessments of protein expression could be made by analysing tumour from different biopsy cores or from tumour at different depths within an individual core. Notably, the H&E and IHC analyses of cross-sections of cores in the Checkerboard TMAs detected cancer in nearly one-half of biopsies originally judged as normal by conventional H&E analyses of the biopsies. Such a finding may in part be due to the improved sensitivity allowed by use of H&E and IHC in combination, and in part achieved by the inherent advantage of this technique in being able to examine a greater depth of tissue. In the current series, this finding did not result in any new cancer diagnosis because all samples were taken from patients who had already been diagnosed with prostate cancer. However, in future studies, it may well be interesting to compare the results obtained in conventional diagnosis using needle biopsy specimens with those obtained from H&E and IHC analyses on cross-sections from Checkerboard TMAs.

Other investigators have reported a 10–30% loss of tissue when constructing conventional TMAs from a large number of cores using manual or automated methods (Schraml *et al*, 1999; Mucci *et al*, 2000; Hoos and Cordon-Cardo, 2001; Mengel *et al*, 2003). This compares to a 9% tissue loss reported in this study. Our low level of loss may indicate that re-embedding the checkers using hot paraffin wax holds the cores more tightly compared to conventional TMA, where the recipient wax block is softened at 36°C to secure the cores.

Needle biopsies provide vital information about the biology and natural history of the cancer (Sotiriou *et al*, 2002) and microarray studies are showing great promise in providing diagnostic as well as prognostic information in a variety of cancers (Pusztai *et al*, 2003). Tissue microarray studies in prostate cancer evaluating predictive markers have, however, been limited to TURP and radical prostatectomy specimens (Bubendorf *et al*, 1999; Dhanasekaran *et al*, 2001; Foster *et al*, 2004). Trans-rectal ultrasound-guided biopsies now predominantly provide diagnostic tissue, but are in general unsuitable for construction of TMA using conventional methods, because of the orientation of the small biopsies. The technique for the production of TMAs from needle biopsy specimens allows formalin-fixed prostatic needle biopsy specimens to be used: (i) in a diagnostic context for assessing multiple IHC markers in patients prior to treatment and (ii) together with both TURP and radical prostatectomy specimens in high-throughput screening for new prognostic markers. The analysis of multiple markers in prostate cancer is important because it may be the analysis of a combination of markers rather than a single marker that provides the best prognostic information (Tricoli *et al*, 2004). This technique may additionally be applicable in analysing needle biopsies taken from other cancer types.
